# Infective larvae of *Cercopithifilaria* spp. (Nematoda: Onchocercidae) from hard ticks (Ixodidae) recovered from the Japanese serow (Bovidae)

**DOI:** 10.1051/parasite/2012001

**Published:** 2013-01-17

**Authors:** Shigehiko Uni, Odile Bain, Hiromi Fujita, Makoto Matsubayashi, Masako Fukuda, Hiroyuki Takaoka

**Affiliations:** 1 Institute of Biological Sciences, Faculty of Science, University of Malaya 50603 Kuala Lumpur Malaysia; 2 Department of Parasitology, Graduate School of Medicine, Osaka City University Osaka 545-8585 Japan; 3 Muséum National d’Histoire Naturelle, Parasitologie comparée, UMR 7205, CNRS 75231 Paris France; 4 Mahara Institute of Medical Acarology Tokushima 779-1510 Japan; 5 National Institute of Animal Health, NARO Ibaraki 305-0856 Japan; 6 Research Promotion Project, Oita University Oita 879-5593 Japan; 7 Department of Infectious Disease Control, Faculty of Medicine, Oita University Oita 879-5593 Japan

**Keywords:** Nematoda, Onchocercidae, *Cercopithifilaria*, Ixodidae, Infective larvae, Japanese serow

## Abstract

Hard ticks taken from the Japanese serow, *Capricornis crispus*, in Yamagata Prefecture, Honshu, harboured infective larvae of onchocercid filariae after incubation from the 22nd to the 158th day. *Haemaphysalis flava* and *H. japonica* contained one to eight filarial larvae; females, males and a nymph of the ticks were infected. The 44 infective larvae recovered were 612–1,370 μm long, and 11 of them, 930–1,340 μm long, were studied in detail. The larvae possessed the morphologic characteristics of the larvae of the genus *Cercopithifilaria*, namely an oesophagus with a posterior glandular part, no buccal capsule and a long tail with three terminal lappets. Five types (A to E) of infective larvae were identified based on the morphologic characteristics. While to date five species of *Cercopithifilaria* have been described from the Japanese serow, a specific identification of the larvae found in this study was generally not possible. Only type E larvae could be tentatively assigned to *Cercopithifilaria tumidicervicata*, as they had a cervical swelling similar to that of the adults of this species. A key for the identification of the five larval types is presented. The study presents circumstantial evidences indicating that *H. flava* and *H. japonica* may transmit *Cercopithifilaria* spp. to Japanese serows. It also suggests the possibility that such filarial larvae will be found in hard ticks anywhere, because *Cercopithifilaria* is distributed worldwide, though this genus generally goes unnoticed, as its microfilariae occur in the skin, not in the blood, of host animals.

## Abstract (fr)

Les tiques récoltées chez les sérows japonais, *Capricornis crispus*, à Yamagata, Honshu, hébergeaient des larves infectantes de filaires Onchocercidae après incubation du 22^ème^ au 158^ème^ jour. *Haemaphysalis flava* et *H. japonica* avaient une à huit larves ; des mâles, des femelles et une nymphe étaient infestés. Les 44 larves infectantes récoltées étaient longues de 612–1,370 μm et 11 d’entre elles, longues de 930 à 1,340 μm, ont été étudiées en détail. Les larves possédaient les caractères morphologiques des larves du genre *Cercopithifilaria*, c’est-à-dire un œsophage avec une portion postérieure glandulaire, pas de capsule buccale et une longue queue avec 3 languettes terminales. Cinq types de larves infectantes (A à E) ont été identifiés sur la base de caractéristiques morphologiques. Bien que cinq espèces de *Cercopithifilaria* aient été décrites jusqu’ici chez le sérow japonais, une identification spécifique des larves trouvées dans cette étude n’a généralement pas été possible. Seules les larves de type E ont été identifiées provisoirement à *C. tumidicervicata* parce qu’elles avaient un renflement cervical comme les adultes de cette espèce. Une clé d’identification des cinq types de larves est présentée. L’étude présente des preuves circonstancielles indiquant que *H. flava* et *H. japonica* peuvent transmettre *Cercopithifilaria* spp. au sérow japonais. Elle suggère la possibilité de trouver partout de telles larves de filaires chez les tiques parce que *Cercopithifilaria* a une distribution mondiale mais passe généralement inaperçu parce que ses microfilaires sont dans la peau, et non dans le sang.

## Introduction

*Cercopithifilaria* Eberhard [17] is one of the 94 genera of the family Onchocercidae (Filarioidea). It was created as a subgenus of *Dipetalonema* Diesing [17] and was soon elevated to generic level [4]. This genus is remarkable for its very large host range and worldwide distribution. The type species was recovered from a cercopithecus monkey in Africa.

Out of a total of 27 nominal species, positively assigned to the genus [9, *C. laemmleri* (Dasgupta *et al*. 1978) excluded] only five are parasites of cercopithecus monkeys [10, 11, 17]. Twelve species are parasites of ruminants [4, 13, 16, 19, 20, 34–36], three of rodents [4, 31], one of lagomorphs [12], one of South American didelphid marsupials [18], one of an Australian marsupial [31] and four of carnivores [2, 7, 25, 32]. All these species have the same morphologic characteristics, such as the very tiny buccal capsule (preoesophageal ring), the oesophagus without glandular part and the extremity of the female tail with three lappets, two lateral and one axial [4], or exceptionally reduced to two processes [17].

Up to now the mitochondrial *Cox*1 and 12S *r*DNA gene sequences of nine species of *Cercopithifilaria* were analysed and congeneric species were clustered together [1, 21]. In addition, two unnamed species and *Cercopithifilaria grassii* (Noè 1907) were identified in European domestic dogs from their microfilariae only, using both morphologic characteristics and molecular analysis; they were grouped with the remaining nominal *Cercopithifilaria* species [27, 28].

Where data are available, the microfilariae of the species of *Cercopithifilaria* seem to inhabit the skin. Life cycles of a few species were elucidated in Africa [8, 29], Europe [14, 25, 26, 38] and Australia [30], from a porcupine, dogs, roe deer and a rat. The larval development of the different species of *Cercopithifilaria* takes place in Ixodidae, which is notable because hard ticks are uncommon intermediate hosts for Onchocercidae. This feature led to the hypothesis that the exceptionally wide geographic distribution and host range of *Cercopithifilaria*, which suggests many host-switches, might have been facilitated by the hard tick vectors and their peculiar way of life; for instance, their passive long distance transportation, the long lifespan and the feeding cycle that often involves several groups of mammals [7, 28, 34].

In Japan, *Cercopithifilaria* is present in the black bear and in the two indigenous ruminants, the sika deer and the Japanese serow, that harbour one, two and five species, respectively [32, 34–36]. To investigate their transmission, hard ticks were collected from these mammals and incubated; filarial larvae were recovered [37]. The present study concerns the larvae from ticks taken from the Japanese serow. A morphologic study is essential to confirm that the infective larvae indeed belong to the genus *Cercopithifilaria* and also to determine if species-specific characteristics are present at the larval stage found in ticks.

## Materials and methods

Sixteen serows (*Capricornis crispus* Temminck, 1845) were killed on Mt. Zao (1,841 m), Yamagata Prefecture, in the northeastern part of Honshu, between April 1998 and July 2001 in accordance with the policies of the Ministry of the Environment, Japan, concerning their conservation and control.

The head with ears, the entire skin of the body with subcutaneous connective tissues and the limbs were shipped refrigerated to the Osaka City University Medical School for examination 1 or 2 days after each animal was killed. Skin snips were made from each serow to determine the presence of filarioids.

*Serow identification number*: Serows examined for taking skin snips, collecting ticks from the skin and dissecting the carcasses were numbered for identification. Ticks collected from the skin of each carcass were kept in small plastic containers (5 cm in diameter and 7 cm high) with small pores to exchange air and with small pieces of wet filter paper. The containers were placed in a large plastic box with wet tissue paper to prevent desiccation and stored in the incubator (20 °C). Ticks were dissected twice a week from 22nd to 158th day following incubation.

*Tick identification number*: Ticks harbouring filarial larvae were numbered for identification when the ticks were dissected. The method of tick dissection: A tick was placed in a drop of saline on a glass slide, cut by the disposable scalpels under a dissection microscope. The nematode larvae taken from ticks were fixed in 2% formalin in saline. Ticks were identified by one of us (H.F.) based on the morphologic characteristics [39].

For morphologic studies larvae were cleared in lactophenol and examined under a compound microscope equipped with a camera lucida. Following Bain & Chabaud [6], particular attention was paid to the caudal extremity, and several ratios were calculated: tail length/width at anus (character 1), larval body length (character 2), oesophagus length/body length (character 3; expressed as a percentage), tail length/body length (character 4; expressed as a percentage). These ratios were used to establish the generic morphometric formulae for the infective larvae of Onchocercidae. The genital primordium was examined either at the level of the oesophagus (females) or posterior to the oesophagus (males). Measurements are given in micrometres.

## Results

Approximately 2,000 ticks were harvested from 16 serows during the study period. Twenty-two ticks harboured filarial larvae, giving an infection rate of 1%. The number of larvae per tick varied from one to eight (mean 2). The infected ticks were *Haemaphysalis flava* Newmann, 1897, of which eight females and six males were infected, and *Haemaphysalis japonica* Warburton, 1908, of which seven females and one nymph were infected (Table 1).

**Table 1.** Measurements of infective larvae of *Cercopithifilaria* spp. recovered from hard ticks collected from the Japanese serow, *Capricornis crispus*.

Specimen no.	1	2	3	4	5	6	7	8	9	10	11
Body length	1,370	1,280	1,120	1,307	1,250	1,340	970	940	1,270	1,190	1,091
Body width (maximum)	21	18	20	24	21	19	22	20	15	18	18
Nerve ring from head	76	77	72	82	65	73	78	60	75	72	86
Excretory pore from head	120	124	110	136	ND	104	110	ND	105	108	ND
Oesophagus length	375	380	292	267	350	330	284	272	330	267	333
Oesophagus/body length (%)	27.4	29.7	26.1	20.4	28.0	24.6	29.3	28.9	26.0	22.4	30.5
Muscular oesophagus length	122	181	138	155	100	150	132	90	138	140	151
Tail length	56	50	69	52	52	57	60	75	75	57	52
Tail/body length (%)	4.1	3.9	6.2	4.0	4.2	4.3	6.2	8.0	5.9	4.8	4.8
Width at anus	15	15	16	15	15	18	16	18	14	14	15
Tail length/width at anus	3.7	3.3	4.3	3.5	3.5	3.2	3.8	4.2	5.4	4.1	3.5
Genital primordium from head	276*	207*	408**	ND	ND	ND	ND	ND	ND	190*	ND
Type of tail tip	A	A	A	A	B	C	C	D	D	E	E
Tick species	H. flava**	H. flava**	H. flava**	H. japonica*	H. flava**	H. flava**	H. flava**	H. flava*	H. japonica***	H. flava**	H. flava*
Incubation period (days)	42	42	42	47	42	42	74	52	54	73	52
Tick ID no.	Y23	Y23	Y23	Y15	Y23	Y23	Y21	Y16	Y18	Y20	Y16
Serow ID no.	YA1	YA1	YA1	YA27	YA1	YA1	YA2	YA5	YA26	YA2	YA5

*Female, **male, ***nymph, ND: not determined, *H*: *Haemaphysalis*. All measurements in micrometres.

Two second-stage larvae were found from ticks: one larva, 337 long and 20 wide, from a tick (Y1: *H. japonica*, female) dissected at day 22 of incubation after collecting from a serow (YA4); the other larva, 326 long and 21 wide, from a tick (Y2: *H. flava*, female) dissected at day 24 of incubation after collection from the serow (YA7). The latter larva was found together with seven infective third-stage larvae (612–867 long and 20–26 wide). A total of 44 infective third-stage larvae were recovered, and their body length ranged from 612 to 1,340. From 38 to 158 days of incubation, all larvae recovered had already developed to the infective third-stage. The 11 larvae studied in detail were recovered from six ticks: four *H. flava* (three males, one female) and two *H. japonica* (one female, one nymph). The ticks were recovered from five serows and the infective larvae were 940–1,370 long (Table 1). Other measurements were width at midbody, 15–24, total oesophagus length, 267–380 and tail length, 50–75.

**Fig. 1.**
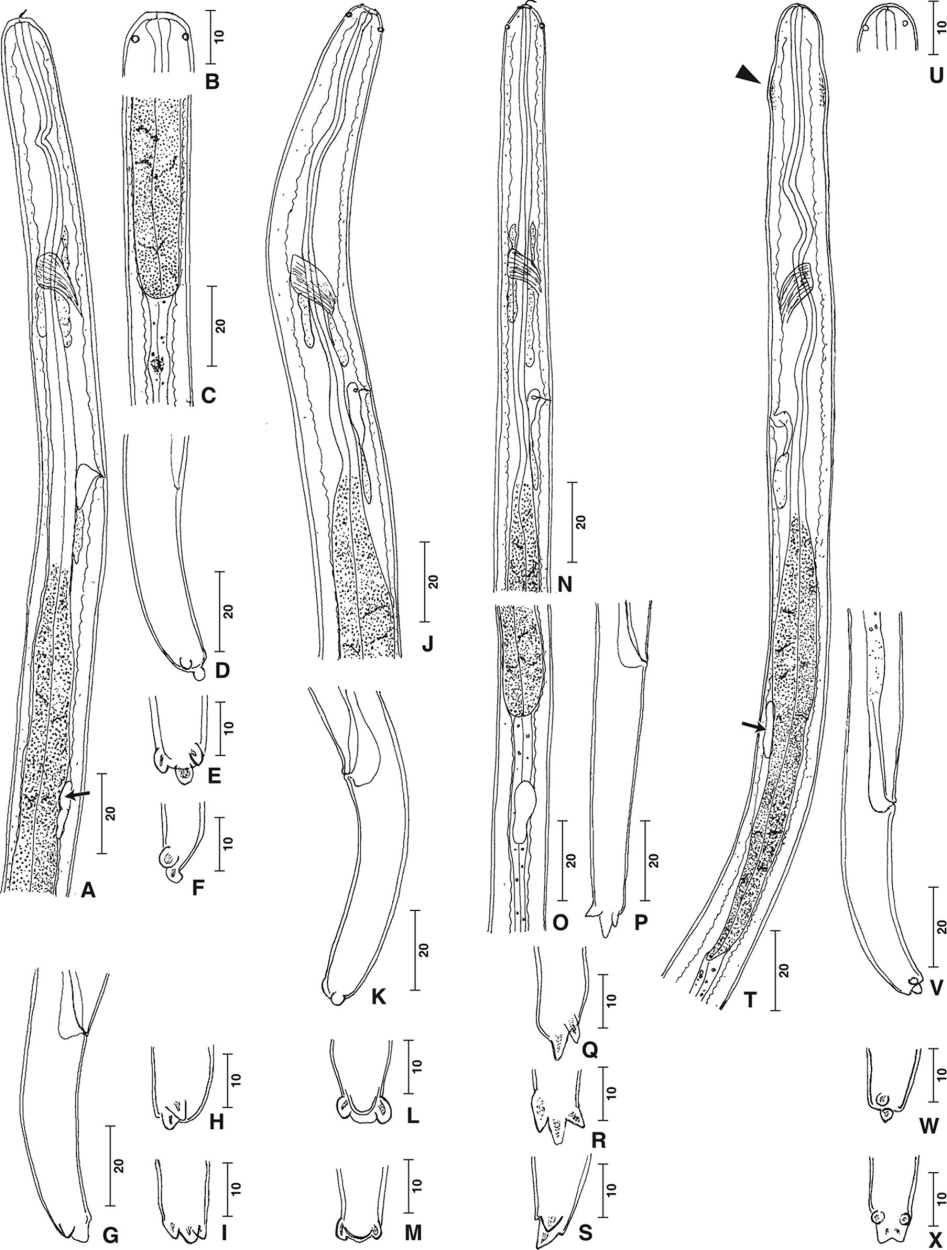
Infective larvae of *Cercopithifilaria* species from ticks collected from the Japanese serow. (A–F) *Type A larva*. A. Anterior part, right lateral view. Female genital primordium, arrow. B. Head. C. Oesophageal-intestinal junction. D. Tail, right lateral view at anus. E. Caudal end, ventral view. F. Caudal end, left lateral view. (G–I) *Type B larva*. G. Tail, right lateral view. H. Caudal end, lateral view. I. Caudal end, ventral view. (J–M) *Type C larva*. J. Anterior part, right lateral view. K. Tail, left lateral view. L-M. Caudal end, ventral view. (N–S) *Type D larva*. N. Anterior part, right lateral view. O. Oesophageal-intestinal junction. *Male genital primordium. P. Tail, right lateral view at anus; ventral view at the end. Q. Caudal end, right lateral view. R. Caudal end, ventral view. S. Caudal end, left lateral view. (T–X) *Type E larva*. T. Anterior part, left lateral view. Cervical swelling, arrowhead; female genital primordium, arrow. U. Head. V. Tail, right lateral view. W. Caudal end, lateral view. X. Caudal end, ventral view. Scale bars: micrometres.

In all larvae the cephalic papillae were easily identified but the buccal capsule was inconspicuous (Figure 1A, B, J, N, U). In some larvae a short sclerotized filament protruded from the mouth (Figure 1A, N). The head was rounded (Figure 1B, J, U) or attenuated (Figure 1N). A cephalic swelling was present in two larvae (Figure 1T, arrowhead). The oesophagus had a muscular anterior part and a glandular posterior part with a mosaic appearance (Figure 1A, C, J, N, T). The glandular part was as long as, or longer than the muscular part but not more than twice as long. The glandular part had a constant width (Figure 1C, O) or was attenuated posteriorly (Figure 1T). The excretory cell with a pore at the posterior group of nerve cells was conspicuous (Figure 1A, J, N, T).

The tail curved ventrally or was straight with its end attenuated (Figure 1G, P) or truncated (Figure 1D, V). The caudal extremity bore two lateral subterminal lappets (named lappets hereinafter) and an axial terminal lappet (named axial point hereinafter). The lappets were rounded (Figure 1D–F, L, M) or conical (Figure 1G–I, P–S). The width at base was equal to the length (Figure 1H, I) or the base was narrower than the lappet length (Figure 1Q, R). The axial point extended from the tail and was conical (Figure 1G, S), or its base was constricted and its shape rounded (Figure 1D–F), or it was slightly divided (Figure 1X), or it was absent (Figure 1K–M); in this case a ventral transverse crest (or boss) was present (Figure 1K). The genital primordium was found at the level of the glandular oesophagus in the female larvae (Figure 1A, T, arrows) and at the level of the posterior part to the oesophageal-intestinal junction in the male larva (Figure 1O, *).

Five morphologic types of infective larvae were identified by the use of the characteristics described above:

**Type A** (Figure 1A–F): Four larvae, nos. 1–4 (Table 1). Body 1,120–1,370 long, 18–24 wide, oesophagus 267–380; head rounded; tail bent ventrally; tip of tail truncated, axial point constricted at base and rounded; lappets rounded.
**Type B** (Figure 1G–I): One larva, no. 5 (Table 1), 1,250 long, 21 wide; head rounded; tail bent ventrally; tip of tail attenuated, prolonged by conical axial point; lappets conical; width at base of lappets and axial point equal to length.
**Type C** (Figure 1J–M): Two larvae, nos. 6 and 7 (Table 1). Body 970 and 1,340, 19 and 22 wide; head rounded; tail bent ventrally; tip of tail without axial point but with ventral transverse crest (or boss); lappets rounded.
**Type D** (Figure 1N–S): Two larvae, nos. 8 and 9 (Table 1). Body 940 and 1,270 long, 15 and 20 wide; head attenuated anterior to cephalic papillae; tail elongated and straight; tip of tail attenuated, prolonged by conical to elongated axial point; lappets conical to elongated.
**Type E** (Figure 1T–X): Two larvae, nos. 10 and 11 (Table 1). Body 1,091 and 1,190 long, both 18 wide; head rounded; cervical swelling; tail bent ventrally; tip of tail truncated, axial point slightly divided, wide in ventral view and narrow in lateral view; lappets small and rounded. The tick (ID no. Y20) harboured the type E larva (specimen ID no. 10) was taken from the serow (YA2) highly infected with *C. tumidicervicata* Uni & Bain, 2001.

To facilitate the identification of the infective larvae from ticks taken from serows, a following key is proposed:

(2) Cervical swelling; caudal axial point divided; small, round lappets; oesophagus attenuated posteriorly.
       Type E larva
(1) Without these characteristics.
(4) Tail 75 μm.
  Caudal axial point and lappets of similar size and conical, elongated shape.
       Type D larva
(3) Tail 50–60 μm.
(6) No caudal axial point, terminal plate with marked ventral crest (or boss), rounded lappets.
       Type C larva
(5) Axial point present.
(8) Axial point constricted at base, of similar shape and size as lappets.
       Type A larva
(7) Axial point conical.
       Type B larva

## Discussion

All infective larvae were approximately 1 mm long and morphologically similar. They possessed an oesophagus with a glandular posterior part, which marked them as belonging to the Onchocercidae [5]. They had a long tail and caudal lappets like *Acanthocheilonema* Cobbold, 1870 [33] and several other closely related genera that had previously been placed in the *Dipetalonema* “lineage” [3, 4, 15] but they lacked the buccal capsule. In this they resembled the infective larvae of the species of *Cercopithifilaria* [6]. The larvae were therefore assigned to the latter genus without any doubt.

However, the morphometric formula established by Bain & Chabaud [6], based on three species parasitic in roe deer, dogs and porcupines, respectively, must be slightly amended. The eight larvae were shorter than 1,300 and three larvae were longer than 1,300 (Table 1); the character 2 (body length): 2B (>1,300) is changed into 2X (X indicates the length between 800 and 1299) and 2B. The characters 1 (tail length/width at anus, 3.0 to 5.8: 1B) and 3 (oesophagus/body length, smaller than 39%: 3A) are confirmed, whereas character 4 (tail/body length, 4.0–5.9%: 4X) is at present 3.9–8.0%: 4X with minor variation and 4B). The original formula, 1B, 2B, 3A, 4X, is therefore changed to 1B, 2X and 2B, 3A, 4X and 4B.

In the set of 11 larvae that were examined in detail, several species seemed to be present since the morphologic characteristics allowed us to distinguish five types of larvae. Attempts to relate each type to one of the five species of *Cercopithifilaria* parasitizing the serows must be made with caution. Firstly, the ticks collected from the serows may contain larvae from other hosts also infected with *Cercopithifilaria*, such as sika deer and black bears [32, 34; ongoing work]. Both *H. flava* and *H. japonica* are three-host ticks that require three kinds of host animals in their life cycle. The larvae of *H. flava* are often found on the skin of hares and the adults parasitize large size mammals such as deer, serows and bears on the Japanese islands, including Okinawa & Hokkaido [22]. The tick also is found in the Russian Far East and China [24]. The larvae and adults of *H. japonica* parasitize wild mammals such as hares, serows, deer and black bears on the western and northern parts of Honshu, Japan [39].

Our study indicates that the ticks, *H. flava* and *H. japonica*, are possible intermediate hosts of *Cercopithifilaria* spp. of serows in Japan; many more larvae were found from *H. flava* than *H. japonica*. We estimate that microfilariae of *Cercopithifilaria* spp. from serows need to develop in a female of *H. flava* to infective stage in 24 days or more at 20 °C. We found that a nymph of *H. japonica* harboured larvae of *Cercopithifilaria* spp. The finding suggests trans-stadial transmission of the filarial larva if a larva had molted into the nymph during incubation.

Secondly, while the caudal extremities of adult females also bear lappets and axial points that differ between species [34–36], the detailed morphology of these structures has to be compared with that seen in infective larvae; similarly to several infective larvae (types A and C), female adults of *C. bulboidea* Uni & Bain, 2001 and *C. shohoi* Uni *et al.*, 1998 present lappets or axial points that are constricted at the base and rounded [35, 36]. While the female adults of *C. minuta* Uni & Bain, 2001 present conical and acute lappets and axial point viewed by a scanning electron microscope [35], the features appear to be similar to those of the type D larva.

Thirdly, the extent of intraspecific variation of minor features of the caudal extremity of infective larvae is at present unknown. Only type E could be tentatively identified as *C. tumidicervicata* based on the features of the anterior part and the tail end. Adults of this species show particular characteristics, such as a cervical swelling, a truncated tail end and a slightly bifid axial point [35]. Such characteristics were found in the type E larvae (Figure 1T, V–X). *Cercopithifilaria tumidicervicata* is found from serows in Yamagata Prefecture, together with *C. shohoi* and *C. minuta* [35]. The larva (specimen ID no. 10) of the type E was found from the tick taken from the serow highly infected with *C. tumidicervicata* (Table 1).

The role of hard ticks in the transmission of *Cercopithifilaria* species and in host-switches during their evolution is once more supported by this study. Several genes of the *Cercopithifilaria* species from the serow and other hosts have been sequenced [1, 21, 27, 28]. The present morphologic analysis will assist in future attempts to identify specimens to species level using gene sequencing, as done by Brianti *et al*. [14] with the *Cercopithifilaria* species of dogs and in the genus *Onchocerca* by Fukuda *et al*. [23].
